# The SAIL Databank: building a national architecture for e-health research and evaluation

**DOI:** 10.1186/1472-6963-9-157

**Published:** 2009-09-04

**Authors:** David V Ford, Kerina H Jones, Jean-Philippe Verplancke, Ronan A Lyons, Gareth John, Ginevra Brown, Caroline J Brooks, Simon Thompson, Owen Bodger, Tony Couch, Ken Leake

**Affiliations:** 1 Health Information Research Unit (HIRU), Centre for Health Information Research & Evaluation (CHIRAL), School of Medicine, Swansea University, UK; 2 Health Solutions Wales (HSW), Brunel House, Cardiff, UK

## Abstract

**Background:**

Vast quantities of electronic data are collected about patients and service users as they pass through health service and other public sector organisations, and these data present enormous potential for research and policy evaluation. The Health Information Research Unit (HIRU) aims to realise the potential of electronically-held, person-based, routinely-collected data to conduct and support health-related studies. However, there are considerable challenges that must be addressed before such data can be used for these purposes, to ensure compliance with the legislation and guidelines generally known as Information Governance.

**Methods:**

A set of objectives was identified to address the challenges and establish the Secure Anonymised Information Linkage (SAIL) system in accordance with Information Governance. These were to: 1) ensure data transportation is secure; 2) operate a reliable record matching technique to enable accurate record linkage across datasets; 3) anonymise and encrypt the data to prevent re-identification of individuals; 4) apply measures to address disclosure risk in data views created for researchers; 5) ensure data access is controlled and authorised; 6) establish methods for scrutinising proposals for data utilisation and approving output; and 7) gain external verification of compliance with Information Governance.

**Results:**

The SAIL databank has been established and it operates on a DB2 platform (Data Warehouse Edition on AIX) running on an IBM 'P' series Supercomputer: Blue-C. The findings of an independent internal audit were favourable and concluded that the systems in place provide adequate assurance of compliance with Information Governance. This expanding databank already holds over 500 million anonymised and encrypted individual-level records from a range of sources relevant to health and well-being. This includes national datasets covering the whole of Wales (approximately 3 million population) and local provider-level datasets, with further growth in progress. The utility of the databank is demonstrated by increasing engagement in high quality research studies.

**Conclusion:**

Through the pragmatic approach that has been adopted, we have been able to address the key challenges in establishing a national databank of anonymised person-based records, so that the data are available for research and evaluation whilst meeting the requirements of Information Governance.

## Background

Vast amounts of personal data are collected routinely by health and social care systems in order to support clinical management and patient care. The secondary use of this data has enormous potential in health-related research, quality improvement, service planning and enhanced clinical decision-making [1]. In the UK, in common with many countries worldwide, the benefits and cost-efficiency potential of using routinely-collected data are widely recognised. The UK Clinical Research Collaboration (UKCRC) is a partnership of organisations working to establish the UK as a world leader in clinical research [2], and this includes a commitment to the use of electronic data within the bounds of data protection and confidentiality [3]. The Health Information Research Unit (HIRU) is an initiative developed by the School of Medicine at Swansea University and it receives core funding as part of the Welsh Assembly Government's commitment to the UKCRC. The aim of HIRU is to realise the potential of electronically-held, person-based, routinely-collected information for the purposes of conducting and supporting health-related research. Furthermore, work is underway to develop a UK Health Informatics Platform under the Office for the Strategic Co-ordination of Health Research (OSCHR) E-Health Records Research Board [4], and the work of HIRU represents the Welsh national component of this system.

In establishing a databank containing person-based data, it is imperative that methods are employed to ensure compliance with the data protection legislation and confidentiality guidelines comprising the NHS Information Governance framework [5] (referred to in this paper as Information Governance), so that the identity of individuals is safeguarded at all stages of the process. Many countries have regulatory and legislative procedures that are of particular relevance to this area of work and the UK is no exception. The Data Protection Act of 1998 [6] enforces the 1995 European Directive on Data Protection [7] and is concerned with data that allow the identification of living persons. This includes not only data that contain clear personal identifiers, such as names, but also data that can indirectly identify an individual [8]. The Caldicott report [9] provides guidelines for the use of patient-identifiable data, and these are of particular importance in National Health Service (NHS) systems. A further safeguard is provided by the National Information Governance Board for Health and Social Care (NIGB) [10] which oversees applications for the common law duty of confidentiality to be set aside in specific circumstances, in accordance with Section 251 of the NHS Act 2006 [11].

A common guiding principle is that person-identifiable data should be anonymised before they are used for secondary purposes. Definitions of data anonymisation vary, but those used within this paper are in accordance with the Medical Research Council (MRC) guidance. This states that: '*anonymised data are data prepared from information from which the person to whom it relates cannot be identified. The term is used when referring to robustly pseudonymised/linked data or unlinked anonymised data*' [12]. For a dataset to be considered truly anonymous, there should be no access within the organisation holding the dataset to any key that could potentially join the anonymised data to a person's identity. Other approaches encrypt confidential identifiable data but allow decryption of the data through the use of a controlled access key. While these processes reduce the risk of identification, the robustness of the anonymisation relies on data management processes to maintain the physical separation of the key from the data within the same organisation.

However, anonymisation alone has been found to be insufficient to address the confidentiality concerns of data holders, in relation to person-based identity and organisational responsibilities. Similarly it cannot fully address the potential for linkage attack in anonymised datasets where researchers may have access to additional information, or where details such as timed-events may be accessed by researchers. A multi-faceted solution including, anonymisation, data aggregation and/or suppression, user authentication, scrutiny to ensure appropriateness of the data use, and disclosure control at analysis and publication, is considered a more robust approach. This must also be combined with responsible data utilisation on the part of the researcher if it is to be effective in data protection. This paper describes the pragmatic multi-level approach taken by HIRU to address the challenges in establishing the SAIL databank for health research and service development within the requirements of the UK Information Governance framework.

## Methods

### Identification of objectives

In order to address the challenges of building the SAIL databank in accordance with Information Governance, a set of objectives was identified. These were to:

1) Ensure data transportation is secure so that data are not lost, or accessed without authorisation, during the acquisition process

2) Operate a reliable record matching process so that accurate anonymised record linkage can be carried out

3) Anonymise and encrypt the commonly-recognised identifiable variables so that identifiable data are not received by HIRU

4) Apply measures to control risk of disclosure in data views created for researchers

5) Ensure data access is controlled and authorised to enable responsible data utilisation

6) Establish methods for scrutinising proposals for data utilisation and approving project outputs to control disclosure risk in results for publication

7) Gain external verification of compliance with Information Governance.

### Creating the databank

Initially HIRU conducted a pilot project in the local authority area of Swansea in West Wales, in partnership with the NHS (primary care general practices and secondary care hospitals) and social services organisations in the area who agreed to provide their datasets. The purpose of the pilot was to develop and refine methods of data extraction, transportation, storage and analysis, to build a prototype solution. It also enabled us to develop a formal process for obtaining permission for data usage from each participating organisation. Central to these developments was the partnership formed with Health Solutions Wales (HSW) which was integral to the design and operation of the anonymisation and matching processes. HSW is an NHS Wales organisation responsible for a wide range of specialist information services including the collection, management, and analysis of data held in a number of national databases [13]. The pilot informed the further development and refinement of technical specifications, data access controls and the establishment of an independent review process for data utilisation, to create an operational databank for research.

External verification of compliance with Information Governance was sought by inviting an independent internal audit to assess the SAIL system. RSM Bentley-Jennison [14] an international company skilled and qualified to provide Information Systems Assurance undertook the work using a key controls audit framework. This was a bespoke audit devised from the Control OBjectives for Information and related Technology (COBIT) [15], the Health Insurance Portability and Accountability Act (HIPAA) [16], the Data Protection Act [6] and the NHS Information Governance toolkit [17]. The audit was conducted via a series of interviews and documentation reviews, plus control checks to test the integrity of the system. These checks were related back to the interviews and were verified against policy documentation, Standard Operating Procedures and computer code in use, to evaluate whether the controls in place were sufficient, and whether they were being followed. The audit employed a number of assessment areas: a) that all input is authorised, complete, accurate and timely; b) that controls exist over access to the system and user rights and permissions; c) the storage and retention arrangements; d) the disposal arrangements; and, e) backup and restoration. A formal report with findings and recommendations for improvement was provided to HIRU following the audit.

We also carried out a wide consultation with government, regulatory and professional agencies on the acceptability of the system. This included: the Department for Public Health and Health Professions (formerly the Department of Health and Social Services Strategy), National Assembly for Wales [18]; the Department for Children, Education, Lifelong Learning and Skills (DCELLS), National Assembly for Wales [19]; the Corporate Health Information Programme (CHIP), National Assembly for Wales [20]; the Office of the Information Commissioner [21]; Informing Healthcare (the national programme for NHS IT in Wales) [22]; the National Research Ethics Service (NRES) [23]; the National Public Health Service for Wales (NPHS) [24]; the British Medical Association (BMA) [25]; the Royal College of General Practitioners (RCGP) [26]; and other professional bodies. These consultations covered a range of issues, including the overall suitability of the proposed system, and its potential to enhance e-health research, to address policy objectives, to ensure data protection and to provide value for money. They also advised on requirements relating to ethical approval for the establishment of the databank and for subsequent research, and on issues of public interest and appropriate data usage. This informed further improvements and resulted in approval for roll-out of the system across Wales. We obtain formal permission for data usage from each participating organisation via their Caldicott Guardians and Information Governance structures.

## Results

### Addressing the objectives

The following is a brief summary of how the objectives were addressed in the development of the databank. Fuller details of the processes underpinning these approaches are described later.

1) Ensuring the security of data transportation was achieved by data providers splitting their datasets at source so that personal data were transported separately from clinical data, and by the use of HTTPS via the NHS Wales National Switching Service for secure data transfers.

2) A matching algorithm (MACRAL) was developed so that reliable anonymised record linkage could be carried out across multiple datasets. Through this method we have achieved consistently efficient matching results with specificity values >99.8%, and sensitivity rates between 95% and 100% for a variety of datasets [27].

3) A process for the anonymisation and encryption of the commonly-recognised identifiable variables was developed, and this is carried out by HSW as a Trusted Third Party (TTP) so that HIRU does not receive any identifiable data. Further data processing at HIRU, including aggregation, masking and second-level encryption provide additional security measures.

4) An algorithm was developed to control the risk of disclosure in data views created for researchers by assessing the level of unique and low-copy number records in the view. This informs further aggregation and/or suppression of particular variables or whole records in the data view.

5) Data access control methods were developed and these operate through a combination of physical and permission-based restrictions and authorisations.

6) A Collaboration Review System, including an independent Information Governance Review Panel, was established to assess the appropriateness of requests to use the data, and of project results before publication.

7) An independent internal audit was commissioned to assess overall compliance with Information Governance and to gain recommendations for improvement.

### Operation of the SAIL system

**Figure 1:**
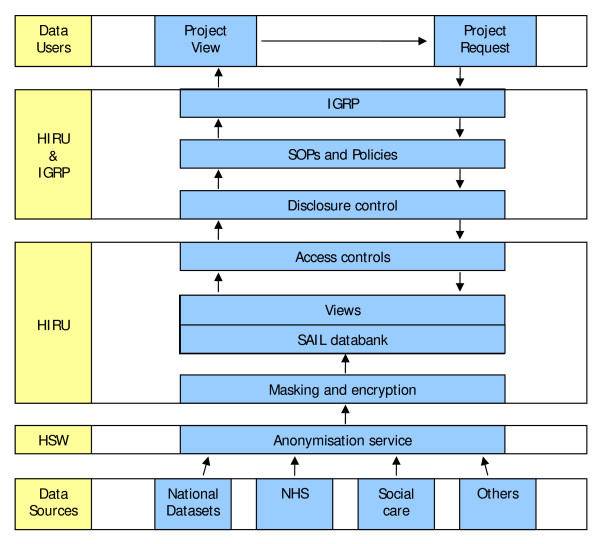
**The SAIL databank system**. This diagram shows the SAIL databank system and the controls in place for data acquisition and utilisation, with an indication of the roles carried out by each party. Beginning at the base of the diagram, HIRU has formal agreements with data providers to provide their data to the databank in accordance with Information Governance. The commonly-recognised identifiers are anonymised at HSW, who provide a trusted third party service to HIRU. Further processes of masking and encryption are carried out at HIRU, and the SAIL databank is constructed. The SAIL databank operates on a DB2 platform (Data Warehouse Edition on AIX) running on an IBM 'P' series Supercomputer: Blue-C. From the top of the diagram, requests to use the data are reviewed by HIRU and an independent Information Governance Review Panel (IGRP) to assess compliance with Information Governance, SOPs and data management policies, and to consider potential disclosure risk, before access can be agreed. Once this is agreed, a data view is created by HIRU technical staff, and access to this view can be made available. For this to happen, further data transformations are carried out to control the risk of disclosure, and the data user signs an access agreement for responsible data utilisation, in accordance with the policies in place and the specifications of the IGRP to comply with Information Governance.

Through the range of approaches adopted, a databank of anonymous person-based records has been successfully established. It operates on a DB2 platform (Data Warehouse Edition on AIX) running on an IBM 'P' series Supercomputer: Blue-C [28]. A diagrammatic representation of the SAIL system and control measures in place is shown in Figure 1. The ways in which the approaches were implemented and now operate are illustrated below by setting out the journey of a dataset from its source to the SAIL databank, and from the databank to utilisation by researchers.

### From data source to databank

HIRU receives anonymised datasets from a wide variety of sources connected with health and well-being so that they can be used in record-linkage research. HIRU supports data providers going through a process of due diligence so they are assured of the robustness of the SAIL system before committing to provide their data. This includes engaging in discussions with the data providers and ensuring they have the opportunity to ask any questions for clarification, and have all the information they require in order to consider providing data to SAIL via their organisational management structures. All data received by HIRU are subject to the process outlined below.

#### At the Data Providing Organisation (DPO)

HIRU has put in place formal documented agreements with DPOs so that the Information Governance requirements of providing the data are met. Initially the DPO identifies from their dataset a system-linking field that can be applied to each individual within their database to act as a unique identifier. This may be an existing field or one generated explicitly for this purpose. The DPO then sorts their data into two distinct categories: demographic data and clinical data. In this context, the term demographic data covers the commonly-recognised personal identifiers: name, address, postcode, date of birth and gender; and clinical data covers data relating to events, diagnoses, measures, interventions and outcomes, etc. When the two data entities have been defined, the DPO generates output data files assigning the unique system-linking field to each individual in the demographic dataset and to each corresponding row in the clinical dataset. The aim of this split-file approach is to enable the removal of the commonly-recognised identifiers from a dataset, and to replace them with a unique, non-identifiable data item, so that data linkage can take place at the individual record level without any identifiable information passing from one organisation to another. The resulting output is two distinct data files each carrying the common key to enable them to be rejoined. These are referred to as File_1 (demographic data) and File_2 (clinical data). A secure HTTPS-based data transportation system was developed to operate across the digital all Wales network and the wider internet, using the NHS Wales National Switching Service (NSS). The NSS utilises secure web services for the transportation layer and end point authentication and authorisation, and controls the onward routing and the workflow under pinning the agreed process. In this way File_1 is transferred to HSW who act as the trusted third party (TTP) for the anonymisation process; File_2 is securely transferred directly to HIRU, thus ensuring that no one party other than the DPO itself ever has access to both demographic and clinical data files.

#### At Health Solutions Wales (HSW)

HIRU works in collaboration with HSW who carry out anonymisation of the demographic data. HIRU and HSW have implemented a series of steps to enable successful anonymisation, namely: matching, encryption, plus De-Identification And Standardisation (DIAS).

The matching process ensures that an anonymised identifier is assigned consistently to each individual in the data file. It relies on the use of the MACRAL (Matching Algorithm for Consistent Results in Anonymised Linkage) algorithm developed by HSW, using the five variables: first name, surname, postcode, date of birth and gender in the matching process. The methodology and accuracy of the matching process is the subject of another paper [27]. Consistent encryption of the output of the matching process, using a method based on the Blowfish algorithm [29], generates a result referred to as the Anonymous Linking Field (ALF). This becomes the individual's unique anonymous identifier and it remains the same for a given individual irrespective of the data source or DPO. The DIAS process takes the remaining fields in the demographic data file and aggregates them into categories, as necessary, to generalise potentially identifying characteristics whilst maintaining a sufficient level of detail to allow meaningful analysis to be conducted. During this phase the postcode is translated into a Lower Super Output Area (LSOA) code (a small area code used by Ordnance Survey [30]), the date of birth is masked using an algorithm to generate a week of birth identifier, and the incoming gender codes are replaced by a standard numerical format. The final stage is to produce an output file containing just the DPO generated system linking field, the categorised data and the Anonymous Linking Field (ALF). This is known as File_3 (anonymised demographics) and it is securely transferred directly to HIRU.

#### At HIRU

HIRU receives data files from DPOs (File_2) and HSW (File_3) and then loads the incoming data into a secure staging area. From here the two files are recombined and built into a normalised base layer, using a bespoke data receipting and handling system, where further processing takes place to ensure integrity and anonymity are maintained. The first process involves the application of an incremental batch number to the entire incoming data set. This batch number serves two main functions. Firstly, it allows all data items for an individual dataset to be identified. This is important when a DPO sends regular data transmissions of new data, and when data from multiple DPOs may reside in a single database table. Secondly, it ensures that the demographic and clinical data can always be joined accurately. By including the batch number as a predicate in the SQL statement alongside the DPO generated system-linking field, the data will always link accurately at an anonymised individual level regardless of how the DPO assigns their system linking field values to individuals over time.

**Figure 2:**
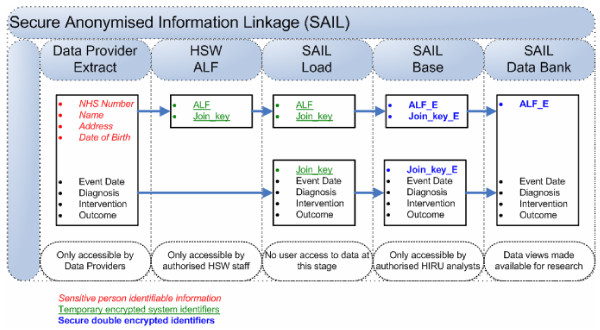
**Stages from data source to databank**. Data providers split their datasets at source into two parts: demographic data (File_1) and clinical data (File_2). File_1 is transferred to Health Solutions Wales for anonymisation and assignment of the Anonymous Linking Field (ALF). This is transferred to HIRU as File_3. File_2 is transferred directly from the data provider to HIRU. A join-key assigned by the data provider allows File_2 and File_3 to be joined at HIRU to build the SAIL databank.

The second process involves the encryption of the HSW generated ALF to produce the ALF-E (Anonymous Linking Field - Encrypted) and of the DPO generated system linking field. This process is known as second level encryption and it ensures analysts from within HIRU and HSW can safely query the SAIL databank without being able to decrypt any data or identify individuals in the DPO's environment through cross-linkage to source datasets. The SAIL databank forms the final layer of the data warehouse, and this is the area that contains the data that are made available for analysis via the creation of data views. Linkage within the SAIL databank is generated through two factors: firstly the encrypted DPO-generated system-linking field which enables the data files provided by a given DPO (File_2 and File_3) to be joined together; and secondly through the ALF-E, which enables datasets across disparate DPOs to be linked at an anonymised individual level. The stages from data source to databank, showing the split-file approach, are illustrated in Figure 2.

#### From databank to utilisation by researchers

Data within the SAIL databank are compiled into views for each individual research project, and these may contain extracts from several datasets, linked anonymously at the individual record level. Before data views are made available to researchers they will have been subjected to further safeguards to ensure anonymity is protected, and access is only granted in accordance with strict controls and authorisations. Requests for data are assessed via a Collaboration Review System (CRS), which comprises review by the HIRU team and by an independent Information Governance Review Panel (IGRP). Review by the HIRU team includes assessing data availability, resource requirements and feasibility of the proposal, and review by the IGRP is to ensure the appropriateness of the request with respect to Information Governance. This is separate to the scientific peer-review process and review by a research ethics committee.

The IGRP was constituted to include representatives from Informing Healthcare [22], the National Research Ethics Service [23], the National Public Health Service for Wales [24], the British Medical Association [25] and Involving People [31]. Together with HIRU, the IGRP assesses each proposal for compliance with Information Governance and with a suite of Standard Operating Procedures (SOPs) and data management policies. The potential for disclosure risk at this stage is considered by the HIRU team and by the IGRP, based on knowledge and custodianship of the datasets, before access can be agreed. The IGRP is provided with a copy of the proposal, completed by the researcher, and members make use of the CRS documentation which sets out the terms of reference and provides proformas for review. The views of the IGRP are open to the members of the panel and to the HIRU team, and are available on request to applicants and other relevant parties with an interest in the particular proposal. Members of the panel are free to seek external advice if additional expertise is required in the review process. The IGRP is empowered to request amendments, or to deny requests, if they find them not in compliance with Information Governance. In this way, although HIRU is the data custodian, there is shared control over access to, and utilisation, of the data.

Further safeguards are needed before the data view is made available to any end user for analysis. Key data characteristics such as the ALF-E and the system-linking field undergo further encryption and the degree of uniqueness within the data view is assessed quantitatively by the NEMO (Numerical Evaluation of Multiple Outputs) algorithm. NEMO is an SQL-based algorithm developed by HIRU that counts the occurrences of unique and low-copy number records in data views prepared for study. It allows the judicious application of further aggregation and/or suppression as necessary to minimise the risk of potential disclosure. It does not automate the process as the specific measures applied depend on the variables requested and the particular project. For example, age may be aggregated into 5 year bands, LSOA codes can be masked, and rare records can be excluded or aggregated into categories. Any proposed amendments that would affect the data view (such as a request for additional variables) or risk potential disclosure are brought to the IGRP, and project outcomes are requested for scrutiny by this panel before publication as a final safeguard.

Data access is subject to stringent controls to ensure that data utilisation complies with Information Governance. Authorised data users are assigned an individual and time-limited logon to access only their data view. Data access is location-specific and only available via designated PCs (secure terminals) which are physically secured to their position and do not have facilities to allow data to be transferred out: they are not internet enabled, and the USB ports and CD drive are disabled. All products of analysis (methodology, scripts, data & results) are securely transferred from these secure terminals to the HIRU data haven, which is an access-controlled drive for authorised users only, using Novell^® ^NetStorage. All statistical outputs are scrutinised by the HIRU team prior to release to researchers. This manual review is carried out by the HIRU senior data analyst and the professor of public health, making use of the Office of National Statistics [32] guidance on disclosure control. Generally, where there are found to be numbers of events less than 5 per unit of analysis, methods are applied to suppress and/or generalise the data. Data users are required to sign an access agreement that they will behave responsibly and comply with all policy and security measures in place before they are provided with the user account. It is worth noting that HIRU staff are also subject to stringent data access conditions. They are also required to sign the data access agreement and to behave responsibly with the data in the course of their work. This includes technical staff and database administrators.

### Independent Internal Audit

The findings of the Information Governance audit were favourable and concluded that the SAIL systems provide adequate assurance that risks material to the work of HIRU are adequately managed and controlled. There were four recommendations for improvement: none were fundamental, two were significant and two merited attention. All the recommendations have been addressed successfully. The areas of assessment were designated a) to e) in the methods section. Under assessment area b) it was recommended (merits attention) that management should ensure that a procedure for the transfer of data from the research terminals to individual PCs is developed and implemented. This was addressed by setting up secure transfer to a data haven on an access-controlled drive (as described above). Under assessment area d) it was recommended (significant) that management should develop a disposal policy as part of the data security framework for the SAIL system. This has been developed and implemented. Under assessment area e) it was recommended that the backup frequency to the data haven should be increased (significant), and that backup tapes for the DB2 database should be stored in a fire-proof safe away from the machine room (merits attention). The backup frequency has been increased with a clear schedule in place, and tapes are stored securely away from the machine room. The database is now backed up to magnetic tapes every evening and the database can be recovered to any point in time during the last two months. The results of the internal audit provided independent assurance to demonstrate that the systems operating in the SAIL databank meet the requirements of Information Governance. We are currently preparing to invite Bentley-Jennison to return to review the changes we have implemented following their recommendations. Audits are to be conducted once a year, but their frequency is also dependent on relevant changes to legislation and regulations and any significant changes we make to our systems. This external scrutiny is important to demonstrate the robustness of the system and to support the confidence of data providers and researchers.

### Datasets incorporated

The SAIL databank already holds over 500 million anonymised person-based records from numerous sources with continual growth in progress. These include data from: Welsh national screening programmes and the cancer registry, registers of births and deaths, national community child health, emergency services, primary care general practices, secondary care hospitals (in-patients, out-patients, day cases, A&E attendances, pathology results) and social care. Many of these datasets are Wales-wide (approximately 3 million population) and further discussions are underway to expand the databank in types of dataset, the range of organisations providing data and increased geographical coverage. As part of this work, datasets on educational attainment are also being included to allow studies of the wider determinants of health and well-being. In addition to these sources of data, the SAIL databank also incorporates datasets generated through primary data collection as part of research studies. These datasets undergo the same processes as datasets from DPOs to ensure anonymity of research participants. This adds high quality detailed datasets in specific areas and, with the potential for anonymous record linkage, it enables long-term follow-up of longitudinal cohorts. A list of the datasets held to date is included in Table 1.

**Table 1:** Datasets held in the SAIL databank

**Dataset**	**Geographical Coverage**	**Data period**	
		
		**From**	**To**
All Wales Injury Surveillance System (AWISS)	Morriston A&E, Swansea Hospitals with A&E departments across Wales	1999 2009	2007 →

General Practice Clinical Systems	Individual practice level across Wales	1993	2008

General Practice Out of Hours Service	Swansea & nearby areas	2005	2008

National Community Child Health Database (NCCHD)	Wales	1987	2007

National Pupil Database	Wales	2003	2008

NHS Administrative Register	Wales	1960	2007

NHS Direct Wales calls	Wales	2006	2008

NHS Hospital admissions (inpatients and day cases)	Wales England	1999 1997	2007 2007

NHS Outpatient appointments	Wales England	2004 2003	2007 2007

Pathology	Morriston Hospital, Swansea Princess of Wales Hospital, Bridgend	2002 1994	2007 2008

Social Services PARIS database	Swansea local authority area	2003	2007

Screening Services	Wales	1990 for cervical screening 1989 for breast screening	Aug 2008 Aug 2008

Welsh Cancer Intelligence and Surveillance Unit (WCISU)	Wales	1990	2006

This table provides a list of the datasets currently held in the SAIL databank. It can be seen that many of the datasets are national in coverage. Where datasets are held only on a regional level at this stage, this is because data collection began with the Swansea area, and plans are underway to extend collection across Wales in due course.

### Utility of the databank

The utility of the databank is demonstrated by increasing engagement in high quality research studies, and we aim to make this resource as available to researchers as possible. Initially HIRU team analysts played an active part in most studies, preparing data views for researchers, but increasingly studies are commencing which include skills and resources for routine data analysis. There are now numerous studies where the data are being used in a variety of ways, and three examples are given here. 1) The data are being used in the development of cohorts in a study funded by the Medical Research Council on ankylosing spondylitis (AS). This project is linking clinical data from rheumatologists (diagnosis, MRI/radiograph images) with existing routinely collected datasets such as the general practice records, hospital data (out-patients clinical data, in-patients, Accident & Emergency, laboratory/pathology data) and social services databases as well as data collected directly from the patients themselves (disease activity, function, quality of life, work limitations). The aim of the study is to develop and characterise a cohort of people with AS in Wales. 2) The data are being utilised to highlight public health improvements, such as through the work of the UK public health centre of excellence (DECIPHer) funded by the UKCRC. The DECIPHer centre studies inter-related issues and takes a multi-disciplinary approach to developing, testing, evaluating and implementing public health interventions. SAIL contributes to this work by linking health and non-health datasets, for example, by providing analyses which use Geographic Information Systems (GIS) to relate the nature of the built environment to body mass index and obesity, by anonymous linkage to general practice data. 3) The data are contributing to the evaluation of national policy, such as the Welsh Assembly Government's Free School Breakfast Initiative (FSBI) funded by the Wales Office of Research & Development. FSBI will include linkage of nutrient and dietary intake data with education databases to explore relationships between diet and factors including: free school meal entitlement, Standard Assessment Tests (SATs) results and General Certificate of Secondary Education (GCSE) performance data; plus linkage to LSOA, census and health and social care databases. The aim is to allow a series of research questions to be addressed to assess the impact of the FSBI on cognitive outcomes and attitudes to diet, including a consideration of social factors.

The continuing development of the SAIL databank is clearly supported by the increasing numbers and types of DPOs who provide their datasets, and of researchers seeking to utilise the SAIL datasets in high quality studies. It is further endorsed by the involvement of national agencies, such as Health Solutions Wales, the National Public Health Service for Wales and Informing Healthcare, who engage with HIRU on continuing developments.

## Discussion

### Observations on the SAIL system

The SAIL databank has been developed through a pragmatic approach, using a range of methods to develop a holistic multi-level solution that combines technical processes, control measures, authorisations and accountability. The decision to establish the SAIL system on a central architecture model, rather than a distributed architecture model, was made because of a number of factors. These were that: many of the existing IT systems in health and social care settings were old and some were unstable; identity management, anonymisation and record-linkage would be far more problematic if attempting to operate in real time; there were unknown issues relating to data quality; there were varying degrees of organisational readiness and resources; and, it was more acceptable to DPOs to provide their anonymous data to SAIL, than for SAIL to plug into their systems and extract data as required. In addition, there were various factors that contributed to the overall rationale for adopting this particular design of central architecture and mode of operation, the main ones being: timeliness; computing capacity; cost; robust data security; reliable matching for accurate record linkage; and, the disparate nature of IT systems in health and social care. Timeliness was a factor of particular importance, as adopting this architecture has enabled the SAIL databank to be established at this time. Other architectures may be possible in the future, but this approach has enabled the creation of an e-health research platform in compliance with Information Governance without further delay, whilst still retaining the opportunity to adopt new methods in the future. Considerable computing capacity is required to accommodate the vast quantities of data. The cost of establishing suitable hardware would have been prohibitive, but for the availability of the Blue-C Supercomputer at Swansea University. In establishing any databank with person-based records, data protection is of paramount importance, and the SAIL databank has been established to be anonymous. This was achieved through the methods described in this paper, via partnership with HSW, so that HIRU does not receive any identifiable data, and so that the data are secure at all stages of the process. The additional measures operated by HIRU are designed to provide further safeguards for data security. A reliable data matching process is essential so that accurate record-linkage can take place. This is mediated by HSW who provide HIRU with the ALF, and this enables reliable record-linkage at the individual level, even across disparate databases from data providers with widely differing types of IT systems. The combination of these factors led to the choice of system that has been established.

It is acknowledged that the individual methods are not necessarily new, but together they provide a system of mutually-reinforcing safeguards to operate the databank in accordance with Information Governance. It is also acknowledged that our approaches could be enhanced and that technical improvements could be made in certain aspects of the work to enhance efficiency, particularly as demand to use the data increases. It has been proposed (in the US) that there should be a national framework for the secondary use of health data [33], and a similar framework is being developed in the UK under the OSCHR E-Health Records Research Board. A structure such as this would most usefully comprise multiple methods to ensure data security and responsible data utilisation, to encompass the full spectrum of risks involved in the re-use of routine data. As well as risks due to data processing, levels of anonymisation, and possible disclosure, issues relating to responsible custodianship, control of the data and transparency would also be addressed to create a holistic governance framework. This sort of multi-faceted approach has been the guiding principle in the development of the SAIL databank to date, whilst recognising the need to continue incorporating further improvements with the rapid developments in the field of health informatics, and to retain the flexibility to respond to possible political changes and new legislation and regulations.

Good working relationships with DPOs are an integral part of the success to date in establishing the databank. This has been motivated by goodwill and the desire to see their data put to good secondary uses within the bounds of Information Governance. Initially some data providers, understandably, had concerns about loss of ownership and control of their data. Opportunities are provided for these issues to be discussed when the DPOs are invited to take part, and all required information is provided to them as part of supporting the due diligence process. It is encouraging that fuller knowledge of the process allays these concerns, as evidenced by the numerous DPOs who have agreed to provide their data to SAIL. There are also additional benefits to DPOs, through the receipt of summary reports on their datasets, which may help them improve data quality and completeness.

### Other challenges

As well as the challenges in establishing the databank in accordance with Information Governance, a significant additional challenge lies in the widely varying data formats and systems in use, with differing semantic and technology architectures, and these can sometimes preclude the process of data acquisition. HIRU is already able to work with many types of dataset, and the Blue-C Super Computer has been fully enabled for GIS data and it supports a variety of data mining techniques. Developing the technologies to enable further disparate types of data to be received is an on-going process for HIRU. Continuing challenges also exist in data quality and consistency, as datasets may be incomplete and coding may be highly variable. These issues must be addressed as they can add significantly to the workload in processing the data and pose considerable challenges for data analysis. Where they cannot be resolved effectively, one solution is to seek to influence the data landscape so that it moves towards using more standardised principles of data management. Such developments towards greater consistency would help address the problem at the data source. This is an important area of work for agencies such as Informing Healthcare and HIRU is actively engaged with them in this process.

In continuing to expand the databank, various factors need to be taken into account to ensure the scalability of the system. Although different architectures may need to be considered in order to improve our systems, we have no anticipated problems with computational capacity for including additional types of dataset on a Wales-wide basis. More likely barriers to scalability are the ultimately limited numbers of new types of DPOs, beyond health and social care, and the organisational issues that may influence agreements to provide data to SAIL. However, success has been achieved in securing data on educational attainment, and the matching process that has been developed (with assignment of the ALF) enables record-linkage across these datasets, as well as those originating in health and social care settings. A similar process has been developed for anonymising residential information, whereby each dwelling is assigned a Residential Anonymous Linking Field (RALF) [34], allowing health-related GIS studies to be conducted. A further consideration in expanding the databank beyond health and social care is of the need for a suitable Trusted Third Party to carry out matching and anonymisation. HSW currently provide this service to HIRU, and as long a suitable gold standard reference dataset is available, HSW would be well-placed to continue this collaboration if more disparate datasets from other sectors are included in the future.

### Future work

Plans for the future development of SAIL are developed in collaboration with the National Assembly for Wales, Informing Healthcare and the IGRP. Future areas of work for HIRU are to further improve our methods, to continue to expand the databank and to increase our engagement in high quality research. HIRU is currently developing a secure Remote Access System for researchers wishing to use the data. Various models for health-related data are described in the literature [35] and we plan to test the functionality and acceptability of different models for the utilisation of the data by the research community. We are also currently developing a method for secure anonymous data transfer to authorised studies, where the appropriate permissions and consent are in place, and the request is approved by the IGRP.

There is considerable recent work in the literature on the development of enhanced quantitative metrics [36,37] and statistical models [38,39] to address disclosure risk in anonymised data made available for users, and we plan to explore the potential of such systems to improve our processing efficiency and our ability to quantify disclosure risk in data views created for researchers. The landscape of health informatics is changing, such that new systems of distributed architecture and federated databases are being promoted and introduced. The UK Health Informatics Platform is to be developed on these principles, and HIRU is engaging in these developments as part of the expansion of the databank. Models for consideration include data grid systems, similar in principle to those that have been established in the field of bioinformatics [40,41]. Another model of interest is MILA (Multi-Institutional Linkage & Anonymisation) which is a technology-independent methodology for the sharing of distributed data, that can be implemented through any suitable combination of transmission and encryption technologies. Explicit demonstrators of this method are the Scottish Family Health Study (SFHS, a national family-based genetics study) [42] and the Scottish Diabetes Research Network [43]. SFHS distributes phenotype, genotype, demographics, identity and routine healthcare data over multiple independent institutions for improved governance. MILA includes an executive authority to oversee the governance of the system as well as technical systems to retrieve and link data from disparate sources.

## Conclusion

The potential for the secondary use of routinely-collected data is widely recognised internationally, including within the UK where it is actively promoted by professional and regulatory bodies. However, appropriate safeguards must be implemented so that these data can be accessed and used for research whilst data protection is upheld. Through the methods that have been developed and implemented, a large-scale and growing databank has been successfully established for research and service development initiatives, whilst complying with the requirements of Information Governance. The volume and breadth of data in the SAIL databank hold great promise as a national resource for research, and as the Welsh component of the developing UK Health Informatics Platform.

## Competing interests

The authors declare that they have no competing interests.

## Authors' contributions

DVF and RAL initiated the concept of the SAIL system. DVF, J-PV and ST designed, developed and implemented the technical systems. TC, KL, GJ and GB designed, managed and carried out the work at HSW. OB developed the NEMO algorithm. CJB managed data access agreements and authorisations, and analysis safeguards. KHJ developed the independent review process and drafted the manuscript. All authors contributed to the manuscript and approved the final version.

## Pre-publication history

The pre-publication history for this paper can be accessed here:


